# Evolutionary Plasticity in Insular Lizard, Adapting over Reproduction, Metabolism, and Color Variation

**DOI:** 10.3390/biology12121478

**Published:** 2023-11-30

**Authors:** Domenico Fulgione, Valeria Maselli, Eleonora Rivieccio, Serena Aceto, Marco Salvemini, Maria Buglione

**Affiliations:** 1 Department of Biology, University of Naples Federico II, 80126 Naples, Italy; fulgione@unina.it (D.F.); serena.aceto@unina.it (S.A.); marco.salvemini@unina.it (M.S.); maria.buglione@unina.it (M.B.); 2 Department of Humanities Studies, University of Naples Federico II, 80138 Naples, Italy; eleonora.rivieccio@unina.it

**Keywords:** *Podarcis siculus*, lizard, plasticity, gene regulation, insularity, transcriptome

## Abstract

**Simple Summary:**

The recent debate on the theory of evolution focuses on various aspects that arise following the evidence of the advancement of knowledge in genetics and molecular biology. In particular, some researchers are inclined to introduce an extended evolutionary theory involving this hypothesis. Many theoretical hypotheses are based on the ever-increasing knowledge of phenotypic plasticity as well as epigenetic mechanisms. In particular, plasticity increases the complexity of the selection–phenotype–genotype interaction, and the collection of cases deriving from empirical experiments helps understand its role. The case of the blue lizard on islets is particularly intriguing due to the speed of its evolution and the modulation of the genome that make these populations a model of plasticity. We have highlighted the adaptive role of regulation of the genome in tissues such as the brain and testis. This would happen because the phenotype adapted to island life is characterized by inter-individual and sexual interaction behaviors that are controlled by genes differentially expressed in these two districts.

**Abstract:**

The Italian wall lizard (*Podarcis siculus*) living on islets exhibits a melanic skin coloration and a suite of adaptive traits lacking in nearby mainland populations. On islets, the unpredictable environmental conditions and highly fluctuating population densities are believed to have produced reversed island syndrome (RIS). Several physiological, behavioral, and life-history changes based on the RIS could result from positive selection on increased activity of melanocortins. We hypothesize that phenotypes on islets are the product of a plastic variation depending on the regulation of specific genes. Focusing on control systems that determine the insular-adapted phenotype, we demonstrated that reproductive markers, involved in the hypothalamus–hypophysis–gonadal axis, and metabolism markers, flags for hypophysis-melanocortin receptors, are all up-regulated in island lizards under the RIS. This behavior, combined with the observed limited variation in the mitochondrial genome, agrees with the hypothesis that plasticity enables populations to persist in novel environmental conditions and that over time, natural selection will “fine-tune” the population to the environment by modifying the phenotype under selection. We believe that analysis of the transcriptome and the single gene expression, such that all the variations observed in the island populations, can be useful to shed light on evolutionary plasticity as a process affecting animals’ populations in general.

## 1. Introduction

**Figure 1:**
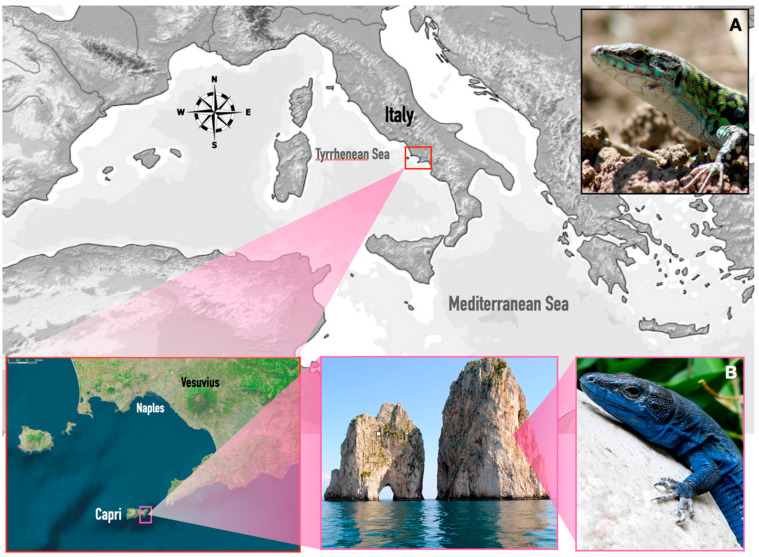
Study area and lizard phenotypes. The insets showed the geographical location of Scopolo, Faraglione of Capri (South Italy). (**A**) Lizard (*Podarcis siculus siculus*) showing a wild-type phenotype on mainland and (**B**) island lizard (*Podarcis siculus coeruleus*) with a blue melanic phenotype on Scopolo. Photos by Domenico Fulgione.

The Italian wall lizard (*Podarcis siculus*) is a lacertid that includes several species endemic to Mediterranean islands [1]. Some of them living on islets sometimes exhibit a clear melanic skin coloration lacking in nearby lizard populations living both on the mainland and on large islands, which generally show a wild-type coloration with a white abdomen and green spots on the dorsal side (Figure 1A,B).

In lizards, morphological and chromatic morphs are rather common [2,3,4], and lizards’ skin coloration is exactly what mainly attracts taxonomists. In the past, in some old investigations, these different chromatic variants were attributed to various, and sometimes bizarre, reasons, such as the Sun’s power, the type of rocks, and diet, linked to the environmental conditions of the islet [5]. Nowadays, a total of 52 subspecies are named [6,7,8]. Some of them live on islets close to the mainland, which houses the nominal species. These divergent phenotypes occur despite the occasional passage of individuals in this narrow stretch of sea, which is highly probable, as suggested by the natural and anthropic materials observable on the facing banks of the island and mainland.

Previous studies on the wall lizards, in addition to the analysis of skin melanization [9], show the variation in a wide range of characteristics, including skull shape and sexual dimorphism [10,11], egg size and plasma dihydrotestosterone levels [12], microbiota [13], and immunocompetence [14], providing more and more evidence to support the hypothesis that insular lizards resort to a precise adaptive strategy, named Reversed Island Syndrome (RIS) [10]. Lizards living on islets close to the mainland exhibit a suite of traits, including higher food intake rates, increased aggressiveness, strong sexual dimorphism, and more resource allocation into reproduction compared to mainland relatives. The features would be the answer to the environmental variations, which can be severe and unpredictable for animals restricted on an islet, affecting population size and determining an unforeseeable mortality schedule. In particular, in this scenario, the individuals investing in reproduction and expressing sexually selected traits should have higher fitness and better survival [15]. Furthermore, shifting efforts from growth towards reproduction may be a winning strategy under resource limitations and/or conspecific competition [16]. We define the theory “RIS” considering that these observed traits are opposite to high population densities, gigantism, decreased fecundity, reduced territoriality, aggressiveness, delayed sexual maturity, and reduced energy expenditure traditionally involved in island syndrome (IS) [17,18,19,20,21].

The phenotypic variations observed under RIS are manifested in a short evolutionary time [22]. Indeed, Buglione and co-workers estimated the time of the coalescent lineage in mainland-island lizard populations using mtGenomes, showing a low mitochondrial mutation rate in island lizards despite the conspicuous phenotypic changes. In addition, transcriptome analyses showed significant differential gene expression between islet and mainland lizard populations [22]. However, these results differ from those of other island lizard populations, suggesting that island-species-specific characteristics can play a relevant role [23].

The knowledge of the molecular and regulatory mechanisms linking environmental influences, functional variation, and the development and evolution of new phenotypes in the wild is still limited [24,25]. Here, we present empirical evidence to corroborate the plasticity hypothesis in island lizards. First of all, we analyze the whole transcriptome of the brain and testis of lizards on the islet and mainland to highlight patterns of expression that differentiate these populations. Then we focus on some indicators of the pathways underlying the behaviors observed in the island lizards, including reproduction, color variations, and metabolism that are generally linked to the melanocortin system as well as to the hypothalamus–hypophysis–gonadal (HHG) axis [26,27,28].

We demonstrate that analysis of the transcriptome and the expression of single genes, such that all the variations observed in the island populations of *P. siculus,* can be helpful to shed light on evolutionary plasticity as a process affecting animal populations in general.

## 2. Material and Methods

### 2.1. Study Area and Samples

In this study, we have considered the nominal population *P. s. siculus* (Rafinesque-Schmaltz 1810), typically characterized by a white abdomen and a green back (Figure 1A) living on the mainland (Punta Campanella, South Italy), and a melanic island population, *P. s. coeruleus* (Eimer 1872), showing a blue coloration on the abdomen and other RIS traits (Figure 1B), endemic to Scopolo Faraglione of Capri (South Italy, approximately 0.76 ha, 40°320 N, 14°150 E). We used two adult male lizards for each population, aged according to snout-vent length (SVL), which is correlated with age [29], and tried to minimize the impact on the populations by using lizards that died accidentally during sampling.

The animals were kept according to the authorization by the Ministry of the Environment and Protection of Land and Sea (also known as MATTM) (prot. 4363/2015). Experimental procedures were approved by the Ethical Committee for Animal Experiments, University of Naples Federico II (ID: 2013/0096988), according to Italian law.

### 2.2. RNA Isolation

In this study, we analyzed RNA-seq data for mainland and island lizards derived from [30].

The RNA was isolated from the brain and testis tissues of island and mainland lizards. RNA-seq libraries were prepared using the TruSeq Stranded mRNA Sample Prep Kits (Illumina, San Diego, CA, USA). Libraries were 2 × 100 paired-end sequenced using an Illumina HiSeq 2500 System (Illumina, San Diego, CA, USA).

### 2.3. Transcriptome Analyses

The quality of raw sequencing reads was evaluated using FastQC software (http://www.bioinformatics.babraham.ac.uk/projects/fastqc/ accessed on 15 September 2023), followed by trimming of low-quality reads and Illumina adapters using Trimmomatic (v0.33) [31]. The high-quality reads were used as input to perform de novo transcriptome assembly using Trinity (v2.1.1) [32], as described in [33].

The quality of the assembled transcriptome was assessed by the BUSCO v3 pipeline [34], and the level of chimeric transcripts was determined by standalone BLASTn analysis using as a reference the *P. muralis* genome dataset [35].

Transcript annotation was performed using Annocript [36].

### 2.4. Differential Gene Expression between Populations

Transcript-level quantification in the testis and brain of each sample was performed by RSEM software [37] and the Bowtie aligner [38], implemented in the Trinity software package. Differential gene expression was conducted using the edgeR software [39], and the Gene Ontology enrichment analysis (*p* < 0.05) was performed using Annocript [33,36].

### 2.5. Verification of Differentially Expressed Genes by Real-Time PCR

To verify the accuracy of the RNA-seq data, we selected the differentially expressed gene encoding the *gonadotropin-releasing hormone II receptor-like* (*GnRH-R2*) and confirmed its expression level by real-time PCR.

We designed primers (Table 1) based on *GnRH-R2* sequences of *P. muralis* and *P. raffonei* (XM_028709494, XM_028709495, XM_053368156, and XM_053368157) obtained from the National Center for Biotechnology Information (NCBI) database.

Starting from 500 ng of total RNA extracted from the brain and testis of the island and mainland *P. siculus* samples, the first-strand cDNA was obtained using the QuantiTect^®^ Reverse Transcription Kit (Qiagen, Germantown, MD, USA) according to the manufacturer’s instructions. PCR and sequencing of the *GnRH-R2* gene fragment were performed to validate gene expression. Additionally, the expression of the housekeeping *actin* gene [9] (Table 1) was determined to check the cDNA integrity and used as a control for its constitutive and stable expression in most cells and tissues.

Gene fragments were amplified by touch-down PCR in a final volume of 20 μL, with 0.2 μL of *Pfu* DNA polymerase (Thermo Scientific, Waltham, MA, USA), 4 μL of 4× Tris buffer with MgCl_2_, 1.6 μL of dNTPs (each dNTP 2.5 μM), 0.2 μL of 50 μM of each primer, and 100 ng of cDNA template under the following conditions: an initial denaturing step of 98 °C for 3 min; 35 cycles of 10 s at 98 °C, 30 s at 55–63 °C, and 1 min at 72 °C; and a final extension step of 5 min at 72 °C. The amplification products from *P. siculus* were gel excised, cleaned up using a QIAquick Gel Extraction Kit (Qiagen, CA, USA), and sequenced. Chromatograms were assembled using the software Geneious Prime 14, and sequences were analyzed with GenBank BLASTn and BLASTx (BLAST, basic local alignment search tool) to confirm the identity of the fragments. All sequence data generated in this study were deposited in GenBank.

Additionally, we performed real-time PCR on *P. siculus* samples in a Rotor-Gene Q cycler (Qiagen, USA) using the QuantiTect SYBR Green PCR Kit (Qiagen, USA). The reaction was prepared in a final volume of 25 μL, with 50 ng of cDNA, 1 mM of each primer, and 12.5 μL of QuantiFast SYBR Green PCR Master Mix (2×). The PCR cycling profile consisted of a cycle at 95 °C for 5 min, 40 three-step cycles at 95 °C for 15 s, at 60 °C for 20 s, and at 72 °C for 20 s. The *actin* gene was used to normalize the relative expression (Table 1). At the end of each test, a melting curve analysis was done (plate read every 0.5 °C from 55 to 95 °C) to determine the formation of the specific products. Each sample was tested and run in technical duplicates.

Relative quantification analysis was conducted using the 2^−(∆∆Ct)^ method (Livak and Schmittgen 2001). Relative mRNA fold change in gene expression was compared, setting y = 1. Statistical significance of the differential expression was assessed using a Kruskal–Wallis test, and data with *p*-values < 0.05 were considered statistically significant.

## 3. Results

Paired-end sequencing of the RNA extracted from the *P. siculus* brain and testis produced 285,765,062 raw reads. After a quality check, we assembled 239,063,200 high-quality reads and generated a reference transcriptome (Table 2).

**Figure 2:**
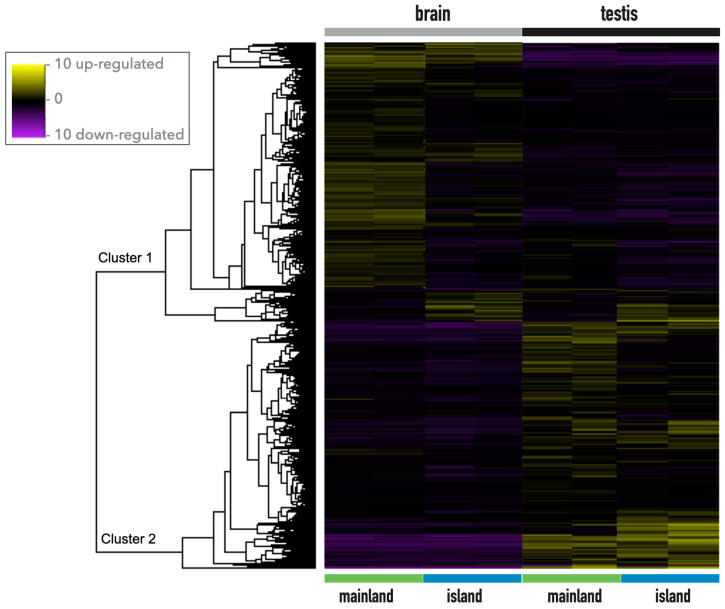
Heatmap of differentially expressed genes in the brain and testis of lizards from mainland and island lizards.

An overall analysis of the gene expression pattern of the brain and testis showed two gene clusters for which a clear difference is observed in the up- and down-regulated genes, both considering population and tissues (Figure 2). Cluster 1 shows generalized up-regulation in the brain, whereas genes of Cluster 2 behave in the opposite way (Figure 2).

**Figure 3:**
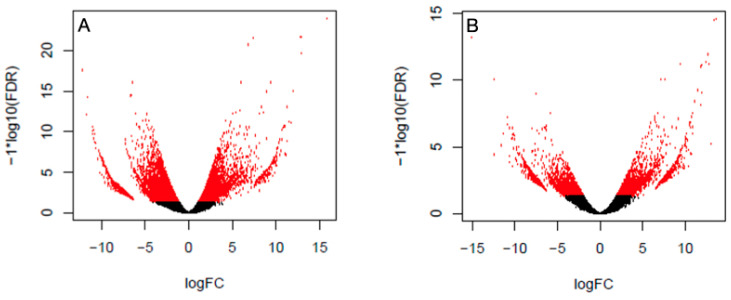
Volcano plot of differentially expressed genes from (**A**) the brain and (**B**) testis of island and mainland lizards. Negative values of logFC (fold changes) represent down-expressed genes, whereas positive values represent up-expressed genes in island samples. Red dots indicate differentially expressed genes with FDR (false discovery rate) < 0.005 and logFC > |2|.

Differentially expressed genes are detected both in brain and testis samples. Considering the latter, the analysis showed 11,778 genes differentially expressed between mainland and island lizards, with 4500 up-regulated and 7278 down-regulated in the island population compared to the mainland population (Figure 3A). The analysis of the testis transcriptome showed the total number of genes differentially expressed between mainland and island lizards to be 7120, with 2283 up-regulated and 4837 down-regulated in the island population compared to the mainland population (Figure 3B).

Among the significantly up-regulated genes (FDR < 0.005, *p* < 0.02) that emerged from analyzing the island brain samples, the GO enrichment revealed functional classes of biological processes (BP) belonging to translation, mitochondrial activity, glucose homeostasis, and transcription regulation. In contrast, among the significantly down-regulated genes (FDR < 0.005, *p* < 0.02), the enriched GO terms belong to cell adhesion, learning, synaptic transmission, and nervous system development. No GO-significantly enriched categories were found among the testis differentially expressed genes.

**Figure 4:**
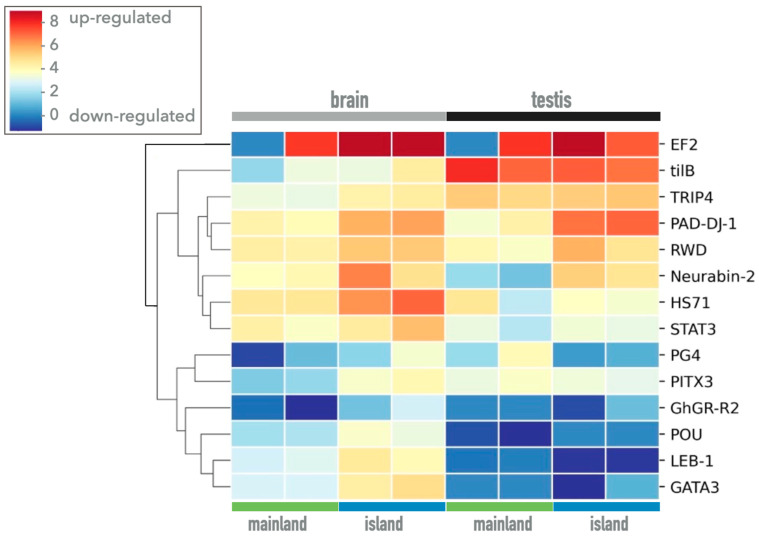
Heatmap of 14 selected genes involved in the hypothalamus–hypophysis–gonadal axis. The gene expression in each sample is reported as log2 of the reads TMM.

To reveal a possible role of the genes involved in the HHG axis in the strategy based on adaptive plasticity, we focused on 14 differentially expressed genes in the brain and testis of island and mainland lizards, as more intuitively shown in the heat map (Table 3 and Figure 4).

The clustering relationship between the populations was close for both tissues, and most of the gene expression patterns between the populations were very different. Some genes that were highly expressed in insular lizards showed low expression levels in the mainland population, and vice versa (Figure 4). All these genes are significantly up-regulated in the brain tissue of the island population, with a logFC ranging from ~2 to 5, while some of them (*TCDD-inducible poly ADP-ribose polymerase*, *protein/nucleic acid deglycase DJ-1*, *neurabin-2*, and *gonadotropin-releasing hormone II receptor*) are also up-regulated in the testis of insular lizards (logFC from ~3 to 7).

The differentially expressed genes detected through in silico and real-time PCR analyses trace a phenotypic profile in agreement with observations on the RIS of island populations.

**Figure 5:**
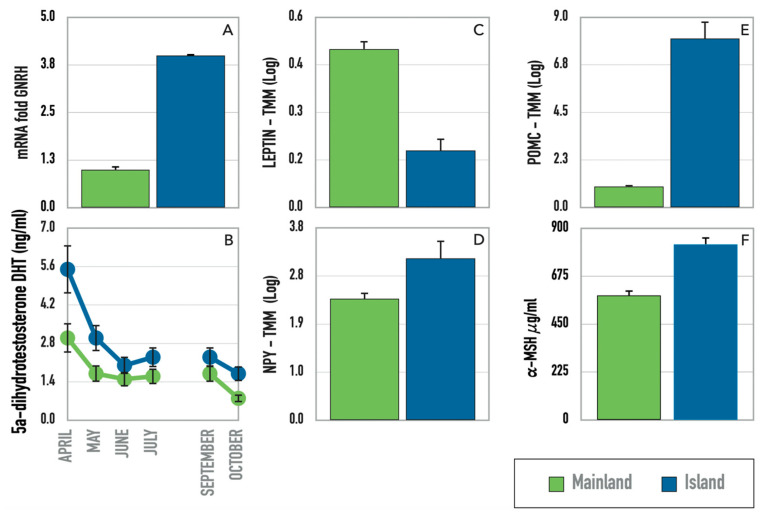
Differential gene expression and plasmatic hormone levels indicative of phenotypic processes that reflect differences between island and mainland lizard populations. (**A**) qRT-PCR of the *GNRH* gene; (**B**) plasma levels of the dihydrotestosterone hormone (modified from [12]); (**C**) differential TMM expression of the gene for *Leptin* in brain; (**D**) differential TMM expression of the *NPY* gene in the brain; (**E**) differential TMM expression of the *POMC* gene in brain; (**F**) plasma levels of *MSH* hormone (modified from [12]).

The reproductive investment, observed in behaviors and hormone levels of insular lizards, can be corroborated by a significant differential expression in the *GnRH-R2* gene (Figure 5A), which is interestingly correlated to higher male dihydrotestosterone levels (Figure 5B), as already assessed in our previous analyses. Following this correlation, we can also interpret the expression of the *Eukaryotic elongation factor 2* gene (*EF2*) that in the testes plays a role in meiosis useful for sperm production (Figure 4), as well as the expression of the *DJ-1* gene, which acts as a regulatory subunit of a 400 kDa RNA-binding protein complex and also modulates androgen receptor-dependent transcriptional activity by binding and blocking transcriptional repressors, such as *PIAS* (*protein inhibitor of activated STAT (signal transducers and activators of transcription*)*xa* and *DJBP* (*DJ-1-binding protein*) [40,41] (Figure 4).

Behavioral observations of hungry and voracious lizards, both in experimental contexts and in the wild, are linked to genes controlling food intake, such as leptin (Figure 5C) and neuropeptide Y (Figure 5D), and also to the melanocortin level. Indeed, the *pro-opiomelanocortin* (*POMC*) gene is differentially expressed in the brain tissue of island and mainland samples, showing a significant logFC 3.7. This indicates that the *POMC* gene is approximately 13-fold more expressed in the brains of island lizards than in those of mainland ones (Figure 5E). Accordingly, the plasma concentration of *MSH* (*melanocyte-stimulating hormone*) is higher in island lizards (Figure 5F).

## 4. Discussion

The debate on phenotypic plasticity inevitably explores the limits of action we give to evolution by natural selection. For this reason, the discussion has been variously articulated, showing opposing points of view: traditional standard evolutionary theory (SET) and extended evolutionary synthesis (EES) [42,43]. There is no need to divide over which of the two processes should be considered predominant, and to appropriately address these topics, it is crucial to collect empirical evidence on species or populations that express plastic variations by defining the magnitude of the selection and the timing of the change [22]. In particular, the phenotypic variations based on the structural changes of the genomes, or on modulations of gene expression, are widely understudied in the evolutionary and ecological genomics of natural populations [44]. From an evolutionary point of view, this leads to a knowledge gap in our understanding of the molecular mechanism of adaptation.

In this scenario, our results are both useful for understanding the involvement of differential expression in natural populations under selection and answering the appropriate question: What are the adaptive traits that respond to natural selection? Indeed, sometimes divergent phenotypes are characterized by a series of more or less related traits that are differently responsible for coping with selection pressure.

Our lizards show phenotypes adapted to insular life and, at the same time, manifest marked skin melanization [9]. The latter, although very striking, does not seem to be a direct response to selection; instead, it is a spandrel [10], which derives from more effective adaptive traits involved in breeding, metabolism, immunity, and growth [10,12,13,14].

In this work, we highlighted how the insular phenotype is regulated by a set of differentially expressed genes and their correlation to reproductive investment and the rapid exploitation of fleeting energetic resources on an island [30].

Focusing on the control systems that determine the insular-adapted phenotype, we demonstrated that the reproductive markers involved in the HHG axis and the metabolism markers—all signals involved in hypophysis-melanocortin receptors—are all up-regulated in insular lizards.

The products that are involved in the pathway of these systems are *GNRH* (which activates the production of sex hormones) [45] and the *POMC* gene, which produces the melanocortins (α-, β-, and γ-*MSH* and *AdrenoCorticoTropic Hormone*) [28]. They are both up-regulated in the island lizards’ brains if compared to the mainland lizards.

Similarly to *GNRH*, over 14 genes involved in the reproductive system are up-regulated in the brain and testis. This pattern is correlated to high testosterone levels during the whole year and to a large inventory of pre-mating reproductive behaviors [10,12].

Differential expression of *POMC*, up-regulated in the island lizards’ brains, can be interpreted considering the aggressive behaviors, pronounced food intake, and melanization of the skin.

The propensity of lacertids to adapt, both plastically and quickly, to environmental modification has been widely observed. For example, *Eremias multiocellata,* exposed to increased temperature variability, produces offspring characterized by higher metabolic and growth rates [46]. Yet, hatchling *Anolis sagrei* in terraria with broad available surfaces, after only 5 months, develops relatively longer hindlimbs compared to lizards living on narrow surfaces [47]. This finding suggests phenotypic plasticity is not related to one specific trait but leads to the production of several phenotypes that are appropriate to particular environments.

Our experimental evidence is particularly important because it defines a set of phenotypical traits useful to face the selective pressure of insular environments. Furthermore, the modulation of these characteristics would make the lizards persistent in the selective insular conditions, waiting for a random mutation that makes their genomes structurally suitable to face the selection [48].

Different known mechanisms act interactively to regulate gene expression in order to keep it in tune with physiological adjustments to the environment [49,50]. Among these, the role of endocrine hormones and DNA methylation has received, and is receiving, special attention in the context of developmental plasticity (see [51,52]).

Our evidence agrees with Baldwin’s hypothesis [53], which affirms that plasticity enables populations to persist in novel environmental conditions and that over time, natural selection will “fine-tune” the population to the environment by modifying the range or average phenotype of the plastic response [53,54,55].

## 5. Conclusions

The populations of small islands, placed close to the mainland, are an extraordinary evolutionary model to investigate the short-term solutions useful to deal with natural selection.

The first formulation of the RIS hypothesis for these populations suggested that, in an unpredictable environment, it was crucial to invest in reproduction and, consequently, in metabolism to anticipate the advent of the next generation. The striking pigmentation is just a consequence of this adaptation. With this contribution, we have provided evidence to better investigate the development of these adaptations.

Here, we highlighted that the hypothalamic–pituitary–gonadal and pituitary-melanocortin pathways were the root of all the adaptive phenotypic variations. Furthermore, the adaptive pathways involving metabolism, reproduction, and color are based on the differential expression of key genes. Although this consideration does not completely clarify this matter, it traces the line of investigation to delve deeper, and also suggests that the phenomenon is based on the species’ plasticity, which will probably be fixed by structural changes. The persistence of lizard populations on small islands recalls the theme of the colonization of new environments due to the plastic properties of the populations. Future investigations may also clarify this intriguing aspect.

## Figures and Tables

**Table 1:** Primer pairs used to test differential gene expression of the selected gene by real-time PCR.

Gene	Primer Name	Sequence Primer 5‘-3‘	Amplicon Size (bp)
*GnRH-R2*	GNRH_1524F	GTCAATGGAAACCCAACGGC	166
	GNRH_1689R	ATGTTGAAGCAGGCGGAAGA	
*Actin*	ACTINA_PS_R	GATCTGGCACCACACCTTCT	104
	ACTINA_PSC_F	TCTTTTCTCTGTTGGCTTTGG	

**Table 2:** Statistics on reference transcriptome.

Transcripts FPKM > 1	373.072
%GC	45.04
Average contig length	821.89
N50 (bp)	1577
Assembled bases (Mbp)	307

**Table 3:** Candidate gene involved in hypothalamus–hypophysis–gonadal axis differentially expressed in the brain and testis of island and mainland lizards.

Gene	Description
*GATA3*	Trans-acting T-cell-specific transcription factor GATA-3
*PAD-DJ-1*	Parkinsonism associated deglycase
*PITX3*	Pituitary homeobox 2-like
*HS71*	Heat shock cognate 71 kDa protein-like isoform X1
*tilB*	Leucine rich repeat containing 6
*GnRH-R*	Type 3/II gonadotropin-releasing hormone receptor
*TRIP4*	Thyroid hormone receptor interactor 4
*STAT3*	Signal transducer and activator of transcription
*EF2*	Eukaryotic elongation factor 2
*RWD*	RWD domain containing 1
*PG4*	platelet glycoprotein 4 isoform X2
*POU*	POU domain protein
*LEB-1*	Isoform 6 of lymphoid enhancer-binding factor 1
*Neurabin-2*	Neurabin-2

## Data Availability

RNA-seq data for mainland and island lizards were deposited in NCBI BioProject PRJNA320862 and PRJNA1041776. Nucleotide sequence was deposited in GenBank under accession number OR818494.
